# A Case for the Conservative Management of Stage IA Cervical Cancer

**DOI:** 10.3390/cancers15205051

**Published:** 2023-10-19

**Authors:** Alexandra Blackman, William Creasman

**Affiliations:** Department of Obstetrics & Gynecology, Medical University of South Carolina, Charleston, SC 29425, USA

**Keywords:** cervical cancer, conservative management, conservative surgery, radical hysterectomy, lymph node metastasis, lymphovascular space invasion

## Abstract

**Simple Summary:**

Cervical cancer remains a major public health concern despite available screening and vaccination. Early-stage disease is frequently treated with radical surgery that has greater operative complications and results in a loss of fertility. Cervical cancer is most frequently diagnosed in pre-menopausal women, making fertility preservation a frequent concern. Additionally, in early-stage disease, there is mounting evidence that radical surgery is not necessary to achieve optimal oncologic outcomes. Using less radical surgical techniques improves patient surgical recovery, avoids long-term complications of surgery, and allows women to retain their fertility if they desire. This editorial presents the data for conservative management of early-stage cervical cancer.

**Abstract:**

Cervical cancer remains a significant public health concern within the United States and across the world. Cervical cancer is most frequently diagnosed in women between the ages of 35 and 44 and therefore affects a younger patient population than many other cancers. The management of early-stage disease has frequently utilized radical hysterectomy with the associated increased surgical morbidity, without clear evidence of any benefits. In stage IA disease, there are retrospective pathologic data supporting the safety of conservative surgery and lymphadenectomy over radical hysterectomy. There are also emerging prospective studies supporting conservative management. This editorial presents the evidence for conservative management of stage IA cervical cancer by reviewing the existing retrospective studies as well as the ongoing prospective studies.

## 1. Introduction

Cervical cancer is the fourth most common cancer in women worldwide, and while screening and vaccination have significantly decreased the number of cases and deaths in the U.S. population, it remains a significant public health concern [1]. The American Cancer Society predicts that in 2023, there will be 13,960 new cases of cervical cancer diagnosed, and 4310 women will die of the disease [2]. With careful adherence to vaccination and screening recommendations, fewer cases of invasive cervical cancer are seen, but in those women who do develop cervical cancer, a trend toward earlier-stage disease should be seen in those patients receiving appropriate preventative care [3].

The diagnosis of early cervical cancer is typically made with a biopsy or on an evaluation of conization specimen. A conization of the cervix is required to make a diagnosis of stage IA cancer. The surgical margins should be tumor-free; otherwise, exact measurements cannot be made. Any gross lesion, irrespective of the depth of invasion, is, by definition, stage IB. Although many advocate radical surgery for some subsets, the data supporting this advocation are somewhat lacking, and management continues to be controversial. The reason for recommending radical hysterectomy in stage IA2 lesions is due to the previously reported increased rate of lymph node metastases (7.4% per Buckley et al.) [4]. When examining this data, many of the reportedly IA lesions were actually found to be IB, as they did not have a cervical cone performed pre-operatively. More recent evidence also brings this recommendation into question, as many studies have shown a low incidence of parametrial involvement in stage IA2 disease.

Cervical cancer is most frequently diagnosed at a younger age compared to other GYN cancers, and concerns such as fertility preservation are more frequently encountered. With many women delaying childbearing and the most frequent ages of diagnosis being between 35 and 44, fertility preservation is often a concern. Radical hysterectomy not only results in a loss of fertility but has significant associated morbidities. More radical dissection and excision can result in bladder and bowel dysfunction as well as sexual dysfunction due to injury to the autonomic nerves in the pelvis. Radical hysterectomy is also associated with higher average blood loss and an increased rate of fistula formation. Reducing treatment-associated morbidity and preserving fertility without threatening oncologic outcomes has become an increasingly important goal for patients and providers alike.

## 2. Historical Perspective

In order to understand the current management of early cervical cancer, it is critical to understand the past classification iterations and the reasons behind the various changes. Mestwerdt observed in 1947 that microscopic invasive cancer of the cervix could be cured with non-radical surgery [5]. The definition of and treatment for microinvasive cervical cancer since this first description have changed dramatically. Over the years, as many as 20 different definitions have been proposed, and as many as 27 terms have been applied to this entity. Microinvasion has been the most frequently used term, but without a finite definition. In 1971, FIGO designated stage IA carcinoma of the cervix as a preclinical carcinoma and described these lesions as “early stromal invasion” [6]. In 1973, the Society of Gynecologic Oncology (SGO) accepted the following statement concerning microinvasion: (1) cases of intraepithelial carcinoma with questionable invasion should be regarded as intraepithelial carcinoma and (2) a microinvasive lesion should be considered when neoplastic epithelium invades the stroma in one or more places to a depth of 3 mm below the base of the epithelium and lymphatic or vascular involvement is not demonstrated [7].

In an attempt to eliminate some of the confusion, FIGO reclassified these lesions in 1973 as stage IA1 and described them as “minimal microscopic evident stromal invasion”. The definition was left vague intentionally, as agreement could not be reached on a more definitive description. Considerable disagreement as to definition was frequently noted in the literature. The Society of Gynecologic Oncology also defined microinvasion as cases of intraepithelial carcinoma with questionable invasion, which should be regarded as intraepithelial carcinoma, and (2) a microinvasive lesion should be redefined as neoplastic epithelium invading the stroma in one or more places to a depth of 3 mm or less without lymphovascular invasion (LVSI).

In 1985, for the first time, FIGO attempted to quantify the histologic definition of stage IA, which was defined as the earliest forms of invasion in which minute foci of invasion are visible only microscopically. Stage IA2 is a macroscopically measured microcarcinoma that should not exceed 5 mm in depth and 7 mm in width. LVSI should not alter staging [8]. As might be expected, this definition was criticized for several reasons. The upper limits for stage IA1 were not defined. This definition could also not be used as a guide for treatment. In 1994, FIGO, in an attempt to better quantify the definition, stated that stage IA was cancer identified only microscopically. All gross lesions, even with superficial invasion, are stage IB cancers. The measured stromal invasion was limited to a maximum depth of 5 mm and no wider than 7 mm. Stage IA1 had measured invasion of stroma up to 3 mm, and stage IA2 had measured invasion of stroma of 3–5 mm and could be no wider than 7 mm [9]. The rationale for the 3–5 mm range with a horizontal spread of <7 mm was based on a three-dimensional volumetric analysis of cervical carcinoma by Burghardt, which revealed that a tumor volume less than 400 mm^3^ was not associated with lymph node metastasis. Using these parameters, the volume would be 367.5 mm^3^, suggesting that lymph node metastatic risk would approach 0% [10]. The depth of invasion should be taken from the base of the epithelium, either surface or glandular, from which it originates. LVSI should not alter the staging. Vascular space involvement was excluded for several reasons, including pathologist disagreement with regards to reproducibility of this entity, concerns of shrinkage artifacts, the number of slides prepared, and studies using special stains to verify true capillary-like space involvement, which identified a discrepancy compared to routine H&E staining. In the ensuing years, multiple studies have been reported with mostly conservative surgery, conization only, or simple hysterectomy for stage IA1, with many authors suggesting the same for stage IA2. Other authors recommend more aggressive surgery for stage IA2 lesions as well as for IA1 with LVSI. In 2018, the International Federation of Gynecology and Obstetrics released an updated cervical cancer staging classification with several changes, including the removal of the 7 mm width as a classification for stage IA disease. The rationale was not based on any data presented by FIGO. Although the definition of stage IA in regard to the depth of invasion has been well accepted since FIGO’s definition in 1985, management remains controversial depending on various risk factors, including lymph node involvement, LVSI, tumor volume, and surgical morbidity, which have all been used as a guide for therapy. The current guidelines recommend modified radical hysterectomy with pelvic lymphadenectomy for both stage IA1 with LVSI and stage IA2 disease.

In addition to the definition debate, management has been more controversial. In early studies, even 1 mm invasion was said to be best managed with radical hysterectomy and pelvic lymphadenectomy. Subsequently, less radical surgery was suggested, although this remains controversial and risk factors have been suggested to guide therapy. The main concern in regard to definitive therapy is whether the disease is limited to the cervix or has metastasized. Potential prognostic factors have been suggested in the hope of identifying that risk.

## 3. Lymph Node Metastasis (LNM)

The risk of lymph node involvement is probably the most concerning factor in determining therapy. Some studies have suggested that as many as 10% of stage IA cancers will have lymph node metastasis. The 1996 study by Buckley et al. has probably been the most quoted as the rationale for more radical surgery in stage IA2 cancers. This was a multi-institutional study of 94 patients collected over 33 years (1961–1993) with a reported rate of lymph node involvement of 7.4%. The authors concluded that patients with stage IA2 disease had a significant risk of LNM and thus should be treated with radical hysterectomy [4]. The methodology of this study was highly criticized by the editorial that accompanied the publication. Of note is that 45% of the patients did not have a conization prior to definitive surgery. The number of hysterectomy specimens with residual disease was not reported. One must assume that the rationale for radical surgery was that a clinical-stage IB lesion was present.

In contrast to the multi-institutional study by Buckley, a well-controlled study of 51 patients with stage IA2 noted no LNM. This was part of a prospective GOG study in which these 51 patients per protocol received a radical hysterectomy with bilateral pelvic lymphadenectomy. A central pathology review was required. All patients required pre-surgical conization with disease limited to the cone and without tumor residuals in the hysterectomy specimen. About a quarter of the patients had LVSI, and none of the patients recurred [11]. Many other studies support the low rate of LNM in stage IA2 disease. A 1994 study by Ostor and Rome conducted a careful clinicopathologic evaluation of 200 patients with micro-invasive disease and found that 22 of these patients had LVSI. Of these patients, 15 underwent nodal dissection, with none being found to have nodal disease [12]. A 2009 review by Rogers and Luesley identified 205 patients with stage IA2 disease and found only 1 to have LNM [13]. In a 2000 study by Elliot et al., 89 patients with stage IA2 disease were identified in their cohort, with only 2 of these having LNM [14]. A 2010 SEER database review by Wright et al. looked at a total of 1409 women who underwent either hysterectomy or conization and found no difference in survival over the course of 5 years [15]. In a review of adenocarcinoma, Bisseling identified 515 stage IA1 and 505 stage IA2 cancers. In patients with stage IA1 disease, the rate of LMN was only 1.1%. In patients with stage IA2 disease, the rate of LNM was 0.7%. Recurrence was similarly low in both stage IA1 and stage IA2 disease, at 1.6% and 1.5%, respectively. No recurrences were noted in the 59 stage IA patients who were treated with conization only [16]. In a review of nine studies of stage IA1 disease, only 0.13% had metastasis to the lymph nodes. The same authors noted that 1.2% of stage IA2 patients had LNM [17].

## 4. Lymphovascular Space Invasion (LVSI)

As noted, FIGO has stated that LVSI should not affect the diagnosis of stage IA1 and IA2 cancers. Nevertheless, many have used LVSI as a guide for more aggressive surgery. The concern about using LVSI as a prognostic factor includes reproducibility. One of the problems with identifying LVSI is that the frequency varies considerably between reports. In a review of eight studies evaluating IA cancers, the incidence varied from 6% to 71%. An interesting study from the Armed Forces Institute of Pathology (AFIP) identified 30 patients with 2–5 mm stromal invasion, and on initial sectioning, 9 (30%) had LVSI. In the 21 patients without LVSI, serial sections were obtained, and 8 had LVSI. A total of 57% of tumors had LVSI. Lymphadenectomies were performed on the 30 patients, and none had LNM [18]. Ostor reviewed 200 cases of stage I cancer, and of the 171 conization specimens, an average of 150 slides per patient were examined. LVSI was suspected in 32 patients using H&E staining but 10 could not be confirmed by ulex europaeus agglutinin I lectin immunoperoxidase staining. Pelvic lymphadenectomy was performed on 58 patients who were all negative for metastases, including 14 (24%) stage IA2 patients associated with LVSI [12]. This has been shown again in a more recent study by Andikyan et al. that noted a high inter- and intra-rater variability in the diagnosis of LVSI [19].

In the same review referenced above by Buchanan of nine studies with stage IA1 disease, 99/1994 (4.9%) had LVSI. Only 13/1033 (0.13%) who underwent pelvic lymphadenectomies had LNM, and 24/2035 (0.1%) developed a recurrence. Of the 12 studies of stage IA2, 70/874 (0.8%) had LVSI, 7/572 (0.01%) had LNM, and 8/892 (0.08%) recurred [17]. Table 1 is modified from Buchanan’s paper with the addition of several newer studies that have been published since.

In a prospective study performed in 2014 by Bouchard-Fortier, 51 patients with stage IA1-stage IB1 disease were managed surgically with simple hysterectomy, cone biopsy with lymph node dissection, or lymph node biopsy. Most patients had stage IA1 disease (55%), but nine of these patients had LVSI and fifteen others had non-SCC histology. In the patients with stage IA2 and IB1 disease, nine of the twenty-three had LVSI. None of the patients in the study recurred [31]. The median follow-up is shorter than in other studies (21 months), but importantly, it suggests that patients with LVSI may be safely included in the cohort of patients eligible for less radical surgery. In a previous study conducted by this same group, low-risk prognostic factors identified included tumor size less than or equal to 2 cm, negative pelvic lymph nodes, and depth of tumor invasion less than or equal to 10 mm. Patients with these low-risk features had a 5-year recurrence-free survival of 96% that was found regardless of LVSI status [32]. In a literature review by Creasman and Kohler, which included 25 studies and evaluating over 6500 patients, LVSI was also not found to be an independent risk factor in early-stage cervical cancer [33]. A recent study by Bogani et al. looked at fertility-sparing treatment with cervical conization and pelvic node dissection in 32 patients with stage IA2, IB1, and IB2 disease. Of these patients, nine had IA2 disease, with 56% of these patients being found to have LVSI. Of these patients, one was found to have a positive lymph node at the time of surgery and required further therapy, but of the patients with negative pelvic nodes, no recurrences were diagnosed in the cohort that received conservative management, with five-year disease-free survival and overall survival being 100% [34].

One of the reasons for the heterogeneous findings of research studies regarding the prognostic significance of LVSI in stage IA disease may be the fact that the majority of studies reporting a significantly increased rate of LNM and recurrence include IB and stage II disease. There is also evidence that the localization and quantification of LVSI impact its predictive value for lymph node metastases and recurrence [35,36].

## 5. Tumor Volume

Tumor volume has been considered the most important prognostic factor for any cancer. Staging is a surrogate for tumor volume. In stage IA, horizontal spread has now been removed from the most recent 2018 FIGO staging without valid explanation. This point was discussed in the 1991 study by Burghardt, which examined 27 patients with small invasive cancers with LVSI who were treated with radical hysterectomy and lymphadenectomy, and found an absence of positive nodes as well as an absence of disease recurrence. The study defined these small invasive cancers as stage IA2 according to the 1985 FIGO classification, with less than 5 mm depth of invasion and less than 7 mm horizontal spread [37]. In a 1992 study by Sevin et al., patients with stage IA2 disease were assessed for risk of LNM. None of the patients included in the study with less than 7 mm of horizontal spread were found to have LNM; however, two patients with 3–5 mm invasion but greater than 7 mm width had LNM [38]. Takeshima noted LNM in 1/29 (3.4%) of patients with stage IA2 (< 7 mm width) but 4/44 (9.1%) if the width was > 7 mm [23].

## 6. Surgical Morbidity

The question of overtreatment is important because, while the lateral extent of resection may only be a small amount of extra tissue, the morbidity of the procedure is much greater than a simple hysterectomy. Short-term complications of increased blood loss, longer operating time, injury to the urologic tract, and neurologic injury are all well documented. Functional disorders of the lower urinary tract are the most common long-term complication of radical hysterectomy, but bowel function can also be affected by interruption of the autonomic nerve fibers during radical hrectomy. In a study by Zullo et al., it was suggested that 70–80% of patients will have some form of urinary complication following radical hysterectomy [39]. Radical hysterectomy involves a much more extensive dissection around the bladder, ureter, and vagina, putting the nerve fibers innervating these structures and running in the areas surrounding them at risk of injury.

Sexual dysfunction is another complication of radical hysterectomy that can result in significant quality of life concerns, and in a Swedish study of 93 women, it affected 25% of women following radical hysterectomy [40]. Additional complications affecting patients undergoing radical hysterectomy include higher intraoperative blood loss and increased risk of lymphedema and lymphocyst formation. With higher risks of intraoperative complications and post-operative morbidity, elucidating a low-risk patient population that would be appropriately managed with less radical surgery would significantly improve the quality of life of early-stage cervical cancer patients, many of whom are young women at the time of surgery.

## 7. Rationale for Conservative Surgery

More recent evidence continues to support the safety of simple hysterectomy for patients with stage IA2 disease. In a large NCDB study published in 2019 by Sia et al., the outcomes for women with stage IA2 and IB1 tumors who underwent simple hysterectomy versus radical hysterectomy were examined, and there was no difference in survival associated with the radicality of the surgery for patients with stage IA2 tumors [41]. In a 2018 study by Chen et al., a total of 101 patients with stage IA2 or IB1 disease were randomized to receive either a radical or simple hysterectomy, with no difference in recurrence rate or 5-year overall survival between the two groups. Morbidity rates, however, were higher in the radical hysterectomy cohort [42]. In a 2021 retrospective study by Liu et al., 440 patients with stage IA2 disease specifically were analyzed; 258 patients underwent radical hysterectomy and 182 patients underwent simple hysterectomy, with both groups having similar 5-year DFS (89.25% vs. 91.14%) and 5-year OS (95.71% vs. 94.76%). When looking at the patient follow-up data in this group, there were six recurrences in the radical hysterectomy group (2.33%) and five recurrences in the simple hysterectomy group (2.75%), with no statistical difference in recurrence between groups [43]. In a large systematic review conducted in 2021 by Wu et al., a total of 21 studies were analyzed for women with stage IA2 as well as stage IB1 disease. A total of 2662 women who underwent simple hysterectomy were included in the study, with the majority of these having stage IB1 disease (61%) and 36.1% having stage IA2 disease. In the studies included in this review, which reported outcomes for both radical and simple hysterectomy, a 4.5% death rate in the radical hysterectomy group and a 5.8% death rate in the simple hysterectomy group were noted. Notably, in patients with stage IA2 disease, there was no significant mortality difference noted between the patients undergoing radical hysterectomy and less radical surgery. The main concern raised in this review is the potentially increased mortality noted in patients undergoing less radical surgery with stage IB1 disease. One important factor to note is that only 72% of the patients undergoing simple hysterectomy underwent lymph node assessment [44]. Lymph node assessment is accepted by some to be a critically important part of surgical assessment, including in cases where less radical surgery would be acceptable.

There is much available evidence to support a more conservative approach to the management of stage IA with LVSI and stage IA2 cervical cancer, and yet we continue to rely on clinical guidelines that recommend more radical surgery for these lower-risk patients. Based on the available data, treating these patients with a simple hysterectomy or cervical conization is a reasonable option that would certainly decrease patients’ surgical morbidity. The main limitation of the previously discussed studies is their retrospective nature. This has spurred the development of multiple prospective clinical trials that have assessed or are currently assessing the safety and efficacy of less radical surgery in early-stage cervical cancer, including the ConCerv Trial, the Less Surgical Radicality for Early Stage Cervical Cancer (LESSER) trial (NCT02613286), the Radical versus Simple Hysterectomy and Pelvic Node Dissection with Low-Risk Early-Stage Cervical Cancer (SHAPE) trial, and the Gynecologic Oncology Group (GOG) 278 Trial. The ConCerv trial looked at stage IA2 (33%) as well as stage IB1 (67%) cervical cancer with tumors less than or equal to 2 cm without LVSI and with a depth of invasion less than or equal to 10 mm. Following conservative therapy, which consisted of either a conization or a simple hysterectomy with lymph node assessment, lymph node metastasis was found to be present in 5% of patients, and residual disease in the hysterectomy specimen was found to occur in 2.5% of patients. Disease recurrence was noted in 3.5% of patients and occurred within 2 years of surgery. Of the five patients with positive lymph nodes, two were stage IA2 and the remainder were stage IB1 [45]. The LESSER trial was a phase II non-inferiority trial comparing the safety and efficacy of simple hysterectomy to modified radical hysterectomy in patients with stage IA2 and IB1 tumors of less than or equal to 2 cm in size. The primary endpoint is 3-year disease-free survival, and the secondary endpoints are 3-year overall survival, surgical morbidity, indication of adjuvant therapy, and quality of life. Preliminary data presented as an oral abstract at SGO 2021 showed similar quality of life measures and post-operative complications but greater length of surgery in the radical hysterectomy arm. Survival outcomes have not yet been reported [46]. The SHAPE trial (NCT01658930) and GOG 278 (NCT01649089) are both still active trials that have not yet reported outcomes.

## 8. Conclusions

The mounting evidence supports a move toward more conservative surgery for stage IA1 with LVSI and stage IA2 cervical cancer. By not subjecting our patients with stage IA disease to radical hysterectomy, we have the opportunity to prevent post-operative morbidity and significantly improve their overall quality of life without increasing the risk of recurrence.

## Figures and Tables

**Table 1:** Studies of Stage IA Disease Recurrence and Lymph Node Metastases.

**Stage IA1**	**LVSI**	**LNM**	**Recurrence**
Rob et al. [20]	3/3		
Pluta et al. [21]	3/3	0/3	0/3
Elliott et al. [14]	47/387	1/121	10/387
Lee et al. [22]	7/174	3/116	2/174
Takeshima et al. [23]	5/297	1/82	0/297
Yoneda et al. [24]	10/10	0/10	
Bisseling et al. [16]	3/515	3/261	6/515
Nam et al. [25]	5/149	0/100	1/149
Reade et al. [26]	9/282	2/209	3/336
Total	99/1994 (4.9%)	13/1033 (0.13%)	24/2035 (0.1%)
**Stage IA2**	**LVSI**	**LNM**	**Recurrence**
Rob et al. [20]	4/10	0/10	0/10
Raju et al. [27]	0/7	0/7	0/7
Smrkolj et al. [28]	12/89	0/46	1/89
Lee et al. [22]	4/28	1/27	0/28
Elliott et al. [14]	47/89	2/59	2/89
Takeshima et al. [23]	7/33	1/29	1/33
Rogers and Luesley [13]	-	1/156	6/205
Yoneda et al. [24]	5/40	2/40	0/40
Bisseling et al. [16]	7/506	2/261	5/506
Reade et al. [26]	15/99	0/90	1/117
Creasman et al. [11]	12/51	0/51	0/51
Pluta et al. [21]	4/11	1/11	0/11
Van Meurs et al. [29]	3/14	0/14	0/14
Obrzut et al. [30]	2/15	0/15	-
Total	122/992 (12.3%)	10/816 (1.2%)	16/1200 (1.3%)
